# Why Do Evaluations of eHealth Programs Fail? An Alternative Set of Guiding Principles

**DOI:** 10.1371/journal.pmed.1000360

**Published:** 2010-11-02

**Authors:** Trisha Greenhalgh, Jill Russell

**Affiliations:** 1 Healthcare Innovation and Policy Unit, Centre for Health Sciences, Barts and The London School of Medicine and Dentistry, London, United Kingdom; 2 Division of Medical Education, University College London, London, United Kingdom

## Abstract

Trisha Greenhalgh and Jill Russell discuss the relative merits of “scientific” and “social practice” approaches to evaluation and argue that eHealth evaluation is in need of a paradigm shift.

**Summary Points**

- We argue that the assumptions, methods, and study designs of experimental science, whilst useful in many contexts, may be ill-suited to the particular challenges of evaluating eHealth programs, especially in politicised situations where goals and success criteria are contested.
- We offer an alternative set of guiding principles for eHealth evaluation based on traditions that view evaluation as social practice rather than as scientific testing, and illustrate these with the example of England's controversial Summary Care Record program.
- We invite *PLoS Medicine* readers to join a debate on the relative merits of “scientific” and “social practice” approaches to evaluation and consider the extent to which eHealth evaluation is in need of a paradigm shift.

## Introduction

Much has been written about why electronic health (eHealth) initiatives fail [1]–[4]. Less attention has been paid to why *evaluations* of such initiatives fail to deliver the insights expected of them. *PLoS Medicine* has published three papers offering a “robust” and “scientific” approach to eHealth evaluation [5]–[7]. One recommended systematically addressing each part of a “chain of reasoning”, at the centre of which was the program's goals [6]. Another proposed a quasi-experimental step-wedge design, in which late adopters of eHealth innovations serve as controls for early adopters [5]. Interestingly, the authors of the empirical study flagged by these authors as an exemplary illustration of the step-wedge design subsequently abandoned it in favour of a largely qualitative case study because they found it impossible to establish anything approaching a controlled experiment in the study's complex, dynamic, and heavily politicised context [8].

The approach to evaluation presented in the previous *PLoS Medicine* series rests on a set of assumptions that philosophers of science call “positivist” [9]: that there is an external reality that can be objectively measured; that phenomena such as “project goals”, “outcomes”, and “formative feedback” can be precisely and unambiguously defined; that facts and values are clearly distinguishable; and that generalisable statements about the relationship between input and output variables are possible.

Alternative approaches to eHealth evaluation are based on very different philosophical assumptions [9]. For example,

- “interpretivist” approaches assume a socially constructed reality (i.e., people perceive issues in different ways and assign different values and significance to facts)—hence, reality is never objectively or unproblematically knowable—and that the identity and values of the researcher are inevitably implicated in the research process [10].
- “critical” approaches assume that critical questioning can generate insights about power relationships and interests and that one purpose of evaluation is to ask such questions on behalf of less powerful and potentially vulnerable groups (such as patients) [11].

## Beyond Questions of Science

Catwell and Sheikh argue that “health information systems should be evaluated with the same rigor as a new drug or treatment program, otherwise decisions about future deployments of ICT in the health sector may be determined by social, economic, and/or political circumstances, rather than by robust scientific evidence” ([6], page 1).

In contrast to this view of evaluation as scientific testing, scholars in critical-interpretivist traditions view evaluation as *social practice*—that is, as actively engaging with a social situation and considering how that situation is framed and enacted by participants [12]–[20]. A key quality criterion in such studies is *reflexivity*—consciously thinking about issues such as values, perspectives, relationships, and trust. These traditions reject the assumption that a rigorous evaluation can be exclusively scientific. Rather, they hold that as well as the scientific agenda of factors, variables, and causal relationships, the evaluation must also embrace the emotions, values, and conflicts associated with a program [19]. eHealth “interventions” may lie in the technical and scientific world, but eHealth dreams, visions, policies, and programs have personal, social, political, and ideological components, and therefore typically prove fuzzy, slippery, and unstable when we seek to define and control them [21].

Kushner observes that “The [positivist evaluation] model is elegant in its simplicity, appealing for its rationality, reasonable in asking little more than that people do what they say they will do, and efficient in its economical definition of what data count” ([18], page 16). But he goes on to list various shortcomings (summarised below), which were illustrated in our evaluation of a nationally stored electronic Summary Care Record (SCR) in England [21],[22]. The SCR was part of a larger National Programme for IT in the National Health Service [23], viewed by many stakeholders as monolithic, politically driven, and inflexible [4],[8].

The first problem with scientific evaluation, suggests Kushner, is that programs typically have multiple and contested goals; hence, no single set of goals can serve as a fixed referent for comparison. An early finding of our evaluation was that the SCR program had numerous goals (e.g., politicians were oriented to performance and efficiency targets, doctors saw the main goal as improving clinical quality in out-of-hours care, and civil liberties lobbyists perceived the program an attempt by the state to encroach on individual privacy) [21].

Second, outcomes are not stable; they erode and change over time and across contexts. In the SCR program, it was originally planned that patients would access their electronic record from home via linked software called HealthSpace, thereby becoming “empowered”. But HealthSpace was subsequently uncoupled from the SCR program because it was deemed “high risk” by civil servants [24].

Third, Kushner suggests, the causal link between process and outcome is typically interrupted by so many intervening variables as to make it unreliable. In the SCR evaluation, we documented 56 such variables—including training, permissions, physical space, technical interoperability, local policies and protocols, professional sanction, and point-of-care consent [21].

Fourth, key characteristics of program success may not be articulated in the vocabulary of outcomes and may not yield to measurement. One such dimension of the SCR program was the variable culture of e-governance across different organisations (e.g., the extent to which it was acceptable for staff to forget their passwords or leave machines “logged on” when going to lunch).

Finally, program learning that leads away from initial objectives threatens failure against outcome criteria. In the SCR program, an early finding was that predefined milestones (e.g., number of records created by a target date) were sometimes counterproductive since implementation teams were required to push forward in the absence of full clinical and patient engagement, which sometimes led to strong local resistance. We recommended that these milestones be made locally negotiable. But because critics of the program interpreted missed milestones as evidence of “failure”, policymakers took little heed of this advice.

## Beyond Variables

“Scientific” evaluation aims to produce statistical statements about the relationship between abstracted variables such as “IT response times”, “resource use”, and “morbidity/mortality” [5]. But the process of producing such variables may remove essential contextual features that are key to *explaining* the phenomenon under study. Controlled, feature-at-a-time comparisons are vulnerable to repeated decomposition: there are features within features, contingencies within contingencies, and tasks within tasks [25].

Expressing findings as statistical relationships between variables may draw attention away from people taking action [20]. In the real world of eHealth implementation, designers design, managers manage, trainers train, clinicians deliver care, and auditors monitor performance; people exhibit particular personality traits, express emotions, enact power relationships, and generate and deal with conflict. Technologies also “act” in their own non-human way: for example, they boot up, crash, transmit, compute, aggregate, and permit or deny access. A statistical approach may produce more or less valid and more or less reliable estimates of effect size (and hence a “robust” evaluation), but “When we enter the world of variables, we leave behind the ingredients that are needed to produce a story with the kind of substance and verisimilitude that can give a convincing basis for practical action” ([20], page 124).

“Substance” (conveying something that feels real) and “verisimilitude” (something that rings true) are linked to the narrative process, which Karl Weick called “sensemaking” [26], which is essential in a multifaceted program whose goals are contested and whose baseline is continually shifting. Collection and analysis of qualitative and quantitative data help illuminate these complexities rather than produce a single “truth”. The narrative form preferred by social scientists for reporting complex case studies allows tensions and ambiguities to be included as key findings, which may be preferable to expressing the “main” findings as statistical relationships between variables and mentioning inconsistencies as a footnote or not at all. Our final SCR report was written as an extended narrative to capture the multiple conflicting framings and inherent tensions that neither we nor the program's architects could resolve [21].

## Beyond “Independence” and “Objectivity”

MacDonald and Kushner identify three forms of evaluation of government-sponsored programs: bureaucratic, autocratic, and democratic, which represent different levels of independence from the state [27]. Using this taxonomy, the approach endorsed by the previous *PLoS Medicine* series [5]–[7] represents a welcome shift from a bureaucratic model (in which management consultants were commissioned to produce evaluations that directly served political ends) to an autocratic model (in which academic experts use systematic methods to produce objective reports that are published independently). But it falls short of the democratic model—in which evaluators engage, explicitly and reflexively, with the arguments exchanged by different stakeholders about ideas, values, and priorities—to which our own team aspired. “Independence” as defined by the terms of autocratic evaluation (effectively, lack of censorship by the state and peer review by other academics who place politics out of scope) pushes evaluators to resist the very engagement with the issues that policy-relevant insights require.

In sum, critical-interpretivist approaches to evaluation have different quality criteria and generate different kinds of knowledge than “scientific” (quasi-experimental) approaches. These differences are summarised in Tables 1 and 2.

**Table 1:** Comparison of Key Quality Principles in Positivist versus Critical-Interpretivist Studies.

Positivist Studies		Critical-Interpretive Studies	
Principle	Explanation	Principle	Explanation
1. Over-arching principle of statistical inference (relating the sample to the population)	Research is undertaken on a sample that should be adequately powered and statistically representative of the population from which it is drawn	1. Over-arching principle of the hermeneutic circle (relating the parts to the whole)	Human understanding is achieved by iterating between the different parts of a phenomenon and the whole that they form
2. Principle of multiple interacting variables	The relationship between input and output variables is affected by numerous mediating and moderating variables, the complete and accurate measurement of which will capture “context”	2. Principle of contextualisation	Observations are context-bound and only make sense when placed in an interpretive narrative that shows how they emerged from a particular social and historical background
3. Principle of distance	Good research involves a clear separation between researcher and the people and organisations on which research is undertaken	3. Principle of interaction and immersion	Good research involves engagement and dialogue between researcher and research participants, and immersion in the organisational and social context of the study
4. Principle of statistical abstraction and generalisation	Generalisablity is achieved by demonstrating precision, accuracy and reproducibility of relationships between variables	4. Principle of theoretical abstraction and generalisation	Generalisability is achieved by relating particular observations and interpretations to a coherent and plausible theoretical model
5. Principle of elimination of bias	Good research eliminates bias through robust methodological designs (e.g., randomisation, stratification)	5. Principle of researcher reflexivity	All research is perspectival. Good research exhibits ongoing reflexivity about how the researchers' own backgrounds, interests, and preconceptions affect the questions posed, data gathered, and interpretations offered
6. Principle of a single reality amenable to scientific measurement	There is one reality which scientists may access, provided they use the right study designs, methods, and instruments	6. Principle of multiple interpretations	All complex social phenomena are open to multiple interpretations. “Success criteria” and “findings” will be contested. Good research identifies and explores these multiple “truths”.
7. Principle of empiricism	There is a direct relationship between what is measured and underlying reality, subject to the robustness of the methods and the precision and accuracy of the instruments	7. Principle of critical questioning	The “truth” is not what it appears to be. Critical questioning may generate insights about hidden political influences and domination. Ethical research includes a duty to ask such questions on behalf of vulnerable or less powerful groups.

Adapted from [10].

**Table 2:** Different Kinds of Knowledge Generated by Different Kinds of Evaluation.

Positivist Evaluations	Critical-Interpretive Evaluations
Focuses on objective methods oriented to the collection of “formal knowledge” as data, thereby producing:	Focuses on naturalistic methods that may capture both formal and informal (tacit, embodied, practical) knowledge, and also co-create learning through dialogue between stakeholders, thereby producing:
• Quantitative estimates of the relationship between predefined input and output variables, and confidence intervals around these	• Map of the different stakeholders and insights into their expectations, values, and framings of the program; illumination of who is accountable to whom
• Deconstruction of “context” to produce quantitative estimates and/or qualitative explanations of the effect of mediating and moderating variables on the relationship between input and output variables	• Problematisation of “success”; insights into the struggle between stakeholder groups to define and judge success and whose voices are dominant in this struggle
• Judgement of the extent to which a program has achieved its original goals and the contribution of different elements in the original chain of reasoning to this	• Illumination of how the eHealth technology exacerbates (or, perhaps, helps overcome) power differentials between different groups (e.g., through differential exposure to surveillance or access to data)
• Statistical generalisation, allowing prediction of how well a particular eHealth technology is likely to work in other contexts and settings	• A rich, contextualised narrative that conveys the multiple perspectives on the program and its complex interdependencies and ambiguities•
• Quantification of how evaluators' formative feedback has influenced outcome	Theoretical generalisation, allowing potentially transferable explanations of the dynamic and reciprocal relationship between macro-, meso-, and micro-level influences
• “Endpoint” knowledge with evaluation methods providing the means to the “end” of producing judgements in a final evaluation report	• Reflections on how formative feedback and the relationship between evaluators and evaluands may have influenced the program, hence advice to future evaluators on how to manage these relationships
• Explanatory and predictive knowledge	• Understanding and illumination

## An Alternative Set of Guiding Principles for eHealth Evaluation

Lilford et al. identify four “tricky questions” in eHealth evaluation (qualitative or quantitative?; patient or system?; formative or summative?; internal or external?) and resolve these by recommending mixed-method, patient-and-system studies in which internal evaluations (undertaken by practitioners and policymakers) are formative and external ones (undertaken by “impartial” researchers) are summative [5]. In our view, the tricky questions are more philosophical and political than methodological and procedural.

We offer below an alternative (and at this stage, provisional) set of principles, initially developed to guide our evaluation of the SCR program [22],[28], which we invite others to critique, test, and refine. These principles are deliberately presented in a somewhat abstracted and generalised way, since they will need to be applied flexibly with attention to the particularities and contingencies of different contexts and settings. Each principle will be more or less relevant to a particular project, and their relative importance will differ in different evaluations.

First, think about your own role in the evaluation. Try to strike a balance between critical distance on the one hand and immersion and engagement on the other. Ask questions such as What am I investigating—and on whose behalf? How do I balance my obligations to the various institutions and individuals involved? Who owns the data I collect? [29].

Second, put in place a governance process (including a broad-based advisory group with an independent chair) that formally recognises that there are multiple stakeholders and that power is unevenly distributed between them. Map out everyone's expectations of the program and the evaluation. Be clear that simply because a sponsor pays for an evaluation it does not have special claim on its services or exemption from its focus [30].

Third, provide the interpersonal and analytic space for effective dialogue (e.g., by offering to feed back anonymised data from one group of stakeholders to another). Conversation and debate is not simply a means to an end, it can be an end in itself. Learning happens more through the processes of evaluation than from the final product of an evaluation report [31].

Fourth, take an emergent approach. An evaluation cannot be designed at the outset and pursued relentlessly to its conclusions; it must grow and adapt in response to findings and practical issues which arise in fieldwork. Build theory from emerging data, not the other way round (for example, instead of seeking to test a predefined “causal chain of reasoning”, explore such links by observing social practices).

Fifth, consider the dynamic macro-level context (economic, political, demographic, technological) in which the eHealth innovation is being introduced [28]. Your stakeholder map and challenges of putting together your advisory group should form part of this dataset.

Sixth, consider the different meso-level contexts (e.g., organisations, professional groups, networks), how action plays out in these settings (e.g., in terms of culture, strategic decisions, expectations of staff, incentives, rewards) and how this changes over time. Include reflections on the research process (e.g., gaining access) in this dataset.

Seventh, consider the individuals (e.g., clinicians, managers, service users) through whom the eHealth innovation(s) will be adopted, deployed, and used. Explore their backgrounds, identities and capabilities; what the technology means to them and what they think will happen if and when they use it.

Eighth, consider the eHealth technologies, the expectations and constraints inscribed in them (e.g., access controls, decision models) and how they “work” or not in particular conditions of use. Expose conflicts and ambiguities (e.g., between professional codes of practice and the behaviours expected by technologies).

Ninth, use narrative as an analytic tool and to synthesise findings. Analyse a sample of small-scale incidents in detail to unpack the complex ways in which macro- and meso-level influences impact on technology use at the front line. When writing up the case study, the story form will allow you to engage with the messiness and unpredictability of the program; make sense of complex interlocking events; treat conflicting findings (e.g., between the accounts of top management and staff) as higher-order data; and open up space for further interpretation and deliberation.

Finally, consider critical events in relation to the evaluation itself. Document systematically stakeholders' efforts to re-draw the boundaries of the evaluation, influence the methods, contest the findings, amend the language, modify the conclusions, and delay or suppress publication.

## Conclusion

eHealth initiatives often occur in a complex and fast-moving socio-political arena. The tasks of generating, authorising, and disseminating evidence on the success of these initiatives do not occur in a separate asocial and apolitical bubble. They are often produced by, and in turn feed back into, the political process of deciding priorities and allocating resources to pursue them [17],[19]. The dispassionate scientist pursuing universal truths may add less value to such a situation than the engaged scholar interpreting practice in context [19],[32].

Differences in underlying philosophical position may lead to opposing quality criteria for “robust” evaluations. Some eHealth initiatives will lend themselves to scientific evaluation based mainly or even entirely on positivist assumptions, but others, particularly those that are large-scale, complex, politically driven, and differently framed by different stakeholders, may require evaluators to reject these assumptions and apply alternative criteria for rigour [33],[34]. The precise balance between “scientific” and “alternative” approaches will depend on the nature and context of the program and probably cannot be stipulated in advance. An informed debate on ways of knowing in eHealth evaluation is urgently needed. We offer this paper to open it.

## Abbreviations

eHealth: electronic health
SCR: Summary Care Record
